# Association of HLA-DRB1 locus with treatment response to abatacept or TNF inhibitors in patients with seropositive rheumatoid arthritis

**DOI:** 10.1038/s41598-024-56987-2

**Published:** 2024-03-21

**Authors:** Soojin Cha, So-Young Bang, Young Bin Joo, Soo-Kyung Cho, Chan-Bum Choi, Yoon-Kyoung Sung, Tae-Hwan Kim, Jae-Bum Jun, Dae Hyun Yoo, Hye-Soon Lee, Sang-Cheol Bae

**Affiliations:** 1 Hanyang University Institute for Rheumatology Research, Seoul, Republic of Korea; 2 Hanyang Institute of Bioscience and Biotechnology, Seoul, Republic of Korea; 3 Department of Rheumatology, Hanyang University Hospital for Rheumatic Diseases, Seoul, Republic of Korea

**Keywords:** HLA-DRB1, Amino acid, Treatment response, Abatacept, TNF inhibitors, Seropositive rheumatoid arthritis, Rheumatic diseases, Rheumatoid arthritis, Genetics

## Abstract

The strongest genetic risk factor for rheumatoid arthritis (RA) has been known as HLA-DRB1 based on amino acid positions 11, 71, and 74. This study analyzed the association between specific HLA-DRB1 locus and treatment response to abatacept or TNF inhibitors (TNFi) in patients with seropositive RA. A total of 374 Korean RA patients were treated with abatacept (n = 110) or TNFi (n = 264). Associations between HLA-DRB1 and treatment response after 6 months were analyzed using multivariable logistic regression. Seropositive RA patients with HLA-DRB1 shared epitope (SE) had a favorable response to abatacept (OR = 3.67,* P* = 0.067) and an inversely associated response to TNFi (OR 0.57, *P* = 0.058) based on EULAR response criteria, but the difference was not statistically significant in comparison to those without SE. In analyses using amino acid positions of HLA-DRB1, a significant association was found between valine at amino acid position 11 of SE and good response to abatacept (OR = 6.46, *P* = 5.4 × 10^–3^). The VRA haplotype also showed a good response to abatacept (OR = 4.56, *P* = 0.013), but not to TNFi. Our results suggest that treatment response to abatacept or TNFi may differ depending on HLA-DRB1 locus in seropositive RA, providing valuable insights for selecting optimal therapy.

## Introduction

Rheumatoid arthritis (RA) is a systemic autoimmune disease caused by both genetic and environmental factors. Many studies have identified over 100 RA susceptibility loci across multiple ancestries^1,2^. The shared epitope (SE) hypothesis suggests that HLA–DRB1 alleles, which share a common amino acid motif at positions 70–74 (QKRAA, QRRAA, and RRRAA), contribute to the susceptibility to RA^3^. The SE has been identified to have a significant association with an increased RA risk and to influence the development of anti-cyclic citrullinated peptides (anti-CCP) antibodies in European and Asian populations^4–6^. In addition, several studies have described associations between HLA-DRB1 non-SE alleles and seropositive RA. Amino acid position 11 of HLA-DRB1 (closely related to position 13), which is not traditionally associated with the SE, has shown the strongest association with seropositive RA risk in both Caucasian and Asian populations^7,8^. These positions are located in the peptide-binding groove of the HLA class II beta chain, alongside positions 71 and 74 of HLA-DRB1, and play a role in antigen presentation to CD4 + T cells.

The use of biologic disease-modifying anti-rheumatic drugs (bDMARDs) has significantly improved RA patients’ clinical outcomes. However, since approximately one-third to half of RA patients still do not achieve a good response to bDMARDs, various efforts are focused on identifying biomarkers that can predict the therapeutic efficacy of each bDMARD. Several studies have investigated the relationship between the anti-CCP and the efficacy of abatacept or TNF inhibitors (TNFi)^9^. A study on a European population found an association between a reduced response to TNFi and the presence of rheumatoid factor (RF) or anti-CCP^10^. A meta-analysis showed that anti-CCP-positive patients are more likely to achieve a good response to abatacept, while not to TNFi, compared to anti-CCP-negative patients with RA^11^.

Recent studies have suggested possible associations between HLA-DRB1 SE and the efficacy of abatacept, but conflicting findings regarding TNFi in RA patients. In Japanese observational studies, RA patients with SE receiving abatacept showed greater efficacy at 24 weeks based on European League Against Rheumatism (EULAR) response than SE-negative patients^12,13^. In a head-to-head study in autoantibody-positive early RA (AMPLE study), SE-positive RA patients receiving abatacept showed greater efficacy compared to those receiving adalimumab^14^. In a European study, SE-positive RA patients receiving adalimumab showed a significant association with low disease activity at week 26, while the efficacy of TNFi in a UK population of RA patients showed no association with SE status^10,15^. Based on the understanding that amino acid positions 11, 71, and 74 of HLA-DRB1 have shown a stronger association with the risk of developing RA compared to SE^7,8^, we hypothesized that treatment response to biologics could potentially be explained by the amino acids or their haplotypes at those specific positions. Therefore, in this study, we investigated the impact of HLA-DRB1 alleles specifically based on amino acid positions 11, 71, and 74 to predict treatment response to abatacept or TNFi in a prospective Korean RA cohort.

## Results

### Patient characteristics

In this study, 110 RA patients were treated with abatacept and 264 RA patients were treated with TNFi [including etanercept (n = 124), adalimumab (n = 92), golimumab (n = 30), and infliximab (n = 18)] due to moderate or high disease activity at enrollment, despite receiving conventional synthetic DMARDs for at least 6 months (Table 1). Among the patients receiving abatacept, the median age of RA onset was 47.0 years, and 58.2% of these patients had not previously received biologics. In the group receiving TNFi, the median age of RA onset was 41.0 years, and 76.9% of these patients had not previously received biologics. The change in DAS28 (ΔDAS28) was calculated as the difference between DAS28 at baseline and at 6 months after treatment. No significant differences were observed between the abatacept and TNFi treatment groups in the median baseline DAS28 [6.4 (5.8–7.0) vs. 6.2 (5.6–6.7), P > 0.05] or the median at 6 months [2.3 (1.6–2.9) in the abatacept-treatment group vs. 2.6 (1.8–3.4) in the TNFi-treatment group, P > 0.05].

**Table 1:** Clinical characteristics of seropositive RA patients receiving abatacept or TNF inhibitors.

		Abatacept (n = 110)^a^
Clinical	RA onset-age	47.0 (35.0–56.8)
	Biologics start age	55.0 (48.0–63.0)
	Female	90.0%
	BMI at baseline	22.2 (19.9–23.7)
	Methotrexate	83.6%
	Biologics naïve	58.2%
	Anti-CCP	93.6%
	RF	89.1%
	DAS28 at baseline	6.4 (5.8–7.0)
	SDAI at baseline	36.4 (28.0–44.7)
HLA-DRB1	Shared epitope^c^	70.9%
	Valine at amino acid position 11	64.5%

		TNF inhibitors (n = 264)^b^
Clinical	RA onset-age	41.0 (30.8–53.0)
	Biologics start age	51.0 (41.8–60.0)
	Female	86.4%
	BMI at baseline	22.0 (19.9–24.0)
	Methotrexate	90.5%
	Biologics naïve	76.9%
	Anti-CCP	92.0%
	RF	92.0%
	DAS28 at baseline	6.2 (5.6–6.7)
	SDAI at baseline	33.0 (27.2–41.4)
HLA-DRB1	Shared epitope^c^	75.0%
	Valine at amino acid position 11	68.6%

*BMI* body mass index, *anti-CCP* anti-cyclic citrullinated peptides antibody, *RF* rheumatoid factor, *DAS28* disease activity score in 28 joints.

^a^Median (interquartile range) and percentage were used for continuous and categorical variables, respectively.

^b^TNF inhibitors [etanercept (n = 124), adalimumab (n = 92), golimumab (n = 30), and infliximab (n = 18)].

^c^The shared epitope of HLA-DRB1 was defined as *01:01, *04:01, *04:04, *04:05, *04:08, *04:10, *10:01, *14:02, or *14:06.

The RA patients carrying HLA-DRB1 SE were associated with production of anti-CCP (*P* = 1.1 × 10^–4^), but not RF, compared to those without SE (Supplementary Table S1). Moreover, valine at amino acid position 11 (Val11) of HLA-DRB1 influenced production of anti-CCP (*P* = 3.4 × 10^–4^) and high anti-CCP titers (> three times the upper limit of normal, *P* = 3.2 × 10^–3^) compared to those without Val11.

### Clinical factors associated with treatment response to abatacept or TNFi in seropositive RA patients

To investigate the factors associated with treatment response to abatacept or TNFi, we divided seropositive RA patients treated with abatacept or TNFi into two groups based on EULAR response criteria: good responders (good response) and poor responders (moderate/non-response). We then analyzed clinical variables associated with good responders. In both abatacept and TNFi groups, treatment response was not associated with RA onset-age, biologics start age, sex, body mass index (BMI), co-treatment with methotrexate, or previous TNFi inefficacy (Table 2).

**Table 2:** Clinical factors associated with good responders to abatacept or TNF inhibitors in seropositive RA patients.

		Good responders^a^	Poor responders^a^	*P*^b^
Abatacept (n = 110)	Number of patients	16	94	
	Female	87.5%	90.4%	1.00
	BMI at baseline	22.2 (21.1–23.2)	22.1 (19.6–24.0)	0.98
	RA onset-age	41.0 (32.5–58.5)	48.0 (37.2–56.0)	0.36
	Biologics start age	47.5 (37.5–61.5)	55.0 (49.0–63.0)	0.06
	Methotrexate	93.8%	81.9%	0.41
	Previous TNFi inefficacy	18.8%	25.5%	0.79
TNF inhibitors (n = 264)	Number of patients	88	176	
	Female	83.0%	88.1%	0.34
	BMI at baseline	22.2 (19.7–23.9)	22.0 (20.0–24.4)	0.98
	RA onset age	43.0 (30.0–52.0)	41.0 (31.0–54.0)	0.63
	Biologics start age	51.0 (41.8–58.0)	51.0 (41.8–60.0)	0.30
	Methotrexate	93.2%	89.2%	0.41
	Previous TNFi inefficacy	3.4%	2.8%	1.00

*BMI* body mass index, *DAS28* disease activity score in 28 joints, *TNFi* TNF inhibitors.

^a^Participants were divided into two groups based on EULAR response criteria: good responders (good response) and poor responders (moderate/nonresponse). Median (interquartile range) and percentage were used for continuous and categorical variables, respectively.

^b^*P* values were estimated using the Wilcoxon rank sum test (continuous variables) and chi square test (categorical variables).

### Associations of HLA-DRB1 SE with treatment response to abatacept or TNFi

We conducted multivariable logistic regression analyses to explore the relationship between HLA-DRB1 SE and treatment response to TNFi or abatacept, while adjusting for RA onset-age and sex. In the TNFi group, we found that SE-positive patients were less likely to be classified as good responders compared to the SE-negative group (OR = 0.57 [0.32–1.02], *P* = 0.058) (Table 3). Conversely, in the abatacept group, a higher proportion of good responders was observed among SE-positive patients, although this finding did not reach statistical significance (OR = 3.67 [0.92–24.71], *P* = 0.067) (Table 4). However, the difference was not statistically significant in comparison to those without SE. We observed a negative trend in the association, although not statistically significant, between the HLA-DRB1 *09:01 allele, which is the second significant risk allele in Korean RA population, and treatment response to abatacept (OR = 0.19 [0.01–1.11], *P* = 0.068) or TNFi (OR = 1.09 [0.61–1.92], *P* = 0.78)^5^.

**Table 3:** Association of shared epitope (SE) and amino acid positions 11, 71, and 74 of HLA-DRB1 with good responders in seropositive RA patients treated with TNF inhibitors.

	HLA-DRB1^a^	OR^b^ (95% CI)	*P*^b^
TNF inhibitors (n = 264)	SE	0.57 (0.32–1.02)	0.058
	SE with Val11	0.82 (0.48–1.41)	0.47
	Val11	0.86 (0.49–1.51)	0.60
	VRA (positions 11, 71, and 74)	0.96 (0.57–1.61)	0.86

*SE* shared epitope, *Val11* valine at amino acid position 11, *VRA* haplotype with valine, arginine, and alanine at amino acid positions 11, 71, and 74.

^a^HLA-DRB1 SE alleles (*01:01, *04:01, *04:04, *04:05, *04:08, *04:10, *10:01, *14:02, *14:06), SE with Val11 (*04:01, *04:04, *04:05, *04:08, *04:10, *10:01), Val11 (*04:01, *04:03, *04:04, *04:05, *04:06, *04:07, *04:08, *04:10, *10:01), and VRA (positions 11, 71, and 74: *04:04, *04:05, *04:08, *04:10, *10:01).

^b^Odds ratios (ORs) and *P* values were estimated by multivariable logistic regression analysis adjusted for RA onset-age and sex.

**Table 4:** Association of shared epitope (SE) and amino acid positions 11, 71, and 74 of HLA-DRB1 with good responders in seropositive RA patients treated with abatacept.

	HLA-DRB1^a^	OR^b^ (95% CI)	*P*^b^
Abatacept (n = 110)	SE	3.67 (0.92–24.71)	0.067
	SE with Val11	**6.46** (1.65–43.17)	**5.4 × 10**^**–3**^
	Val11	**5.17** (1.30–34.84)	**0.017**
	VRA (positions 11, 71, and 74)	**4.56** (1.35–21.01)	**0.013**

*SE* shared epitope, *Val11* valine at amino acid position 11, *VRA* haplotype with valine, arginine, and alanine at amino acid positions 11, 71, and 74.

Significant values are given in bold.

^a^HLA-DRB1 SE alleles (*01:01, *04:01, *04:04, *04:05, *04:08, *04:10, *10:01, *14:02, *14:06), SE with Val11 (*04:01, *04:04, *04:05, *04:08, *04:10, *10:01), Val11 (*04:01, *04:03, *04:04, *04:05, *04:06, *04:07, *04:08, *04:10, *10:01), and VRA (positions 11, 71, and 74: *04:04, *04:05, *04:08, *04:10, *10:01).

^b^Odds ratios (ORs) and *P* values were estimated by multivariable logistic regression analysis adjusted for RA onset-age and sex.

### Associations of amino acid positions 11, 71, and 74 of HLA-DRB1 with good responders in seropositive RA patients treated with abatacept or TNFi

We conducted further analyses to determine whether amino acid positions 11/13, 71, and 74 of HLA-DRB1, the strongest RA risk factor, influence treatment response to abatacept or TNFi among seropositive RA patients. We excluded consideration of amino acid position 13 in HLA-DRB1 due to its strong linkage disequilibrium (LD) with position 11^8^. Intriguingly, patients with Val11 of HLA-DRB1 SE exhibited a more favorable response to abatacept (OR = 6.46 [1.65–43.17],* P* = 5.4 × 10^–3^) (Table 4, Supplementary Table S2). Interestingly, RA patients with Val11 also showed a good response to abatacept, regardless of SE (OR = 5.17 [1.30–34.84], *P* = 0.017). However, no significant associations were observed between SE with Val11 or HLA-DRB1 Val11 and good responders in seropositive RA patients treated with TNFi (OR = 0.82 [0.48–1.41], *P* = 0.47, OR = 0.86 [0.49–1.51], *P* = 0.60, respectively) (Table 3).

Next, we investigated whether the haplotypes based on amino acid positions 11, 71, and 74 were associated with good responses. We observed that the valine arginine alanine (VRA) haplotype at amino acid positions 11, 71, and 74 showed a significant association with a good response in seropositive RA patients treated with abatacept (OR = 4.56 [1.35–21.01], *P* = 0.013) (Table 4, Supplementary Table S2). However, no significant association was observed between the VRA haplotype and treatment response to TNFi (OR = 0.96 [0.57–1.61], *P* = 0.86) (Table 3). The association between the VRA haplotype and a good response was observed, not within the SE (Supplementary Fig. S1) in patients treated with abatacept. This suggests that patients with the VRA haplotype were more likely to exhibit a favorable response compared to those with the SE.

## Discussion

This study investigated an effect of HLA-DRB1 on treatment response to abatacept or TNFi in seropositive RA patients. We demonstrated that Val11 of HLA-DRB1 might predict good treatment response to abatacept in seropositive RA patients. Our analysis further revealed a significant association between a good response to abatacept and SE with Val11 (*P* = 5.4 × 10^–3^), as well as the VRA haplotype at amino acid positions 11, 71, and 74 of HLA-DRB1 (*P* = 0.013).

Since a considerable proportion of patients with bDMARDs still do not experience a favorable response, the need for research on predictive biomarkers that can facilitate optimal bDMARDs selection has been consistently emphasized.

We demonstrated a positive association between SE and abatacept response in a Korean seropositive RA population, consistent with previous European and Japanese studies^12–14^, and added a novel result by revealing the effect of HLA-DRB1 amino acid positions on treatment response, which offer greater explanatory power than SE.

In our study, RA patients with SE treated with TNFi showed a less favorable treatment response compared to those with SE-negative. At the amino acid level, we did not observe any significant association between Val11 or the VRA haplotype (at amino acid positions 11, 71, and 74) and treatment response to TNFi. Since a European study suggested a weak association between Val11 and a good EULAR response (OR = 1.14, *P* = 0.04)^16^ and previous studies regarding the association of SE with response to TNFi showed conflicting findings, further research is needed to investigate whether the specific HLA-DRB1 alleles or haplotypes could predict the response to TNFi or not.

Despite our study’s significant findings, there are several limitations. Although we can get statistically significant results even in a relatively small sample sized cohort, it is still necessary to conduct large-scaled studies to confirm our findings. The VKA haplotype (HLA-DRB1 *04:01) is uncommon in the Korean population but prevalent in European populations, necessitating additional studies to verify our findings across different populations. Lastly, both the abatacept and TNFi treatment groups in the study included patients who had previously failed on other bDMARDs. As a result, the proportion of good responders may be relatively lower compared to the group of patients who are naïve to bDMARDs. Also, we did not directly compare the effects of abatacept and TNFi treatments, as the two groups have distinct backgrounds. However, this may actually better reflect real-world data.

Our findings suggest that HLA-DRB1 alleles carrying Val11 may serve as predictive biomarkers for a favorable treatment response in seropositive RA patients receiving abatacept, but not in those receiving TNFi. These results indicate the potential clinical utility of using these biomarkers to guide the selection of optimal therapies for individual RA patients.

## Methods

### Patients

A total of 374 seropositive RA patients who were treated with abatacept (n = 110) or TNFi (n = 264) and fulfilled the 1987 revised American College of Rheumatology (ACR) or 2010 ACR/EULAR criteria were enrolled from Hanyang University Hospital for Rheumatic Diseases. We obtained clinical data including autoantibody profiles (RF, anti-CCP). The Disease Activity Score in 28 Joints (DAS28) was assessed by analyzing the 28-tender joint count (TJC), 28-swollen joint count (SJC), patient global assessment (PtGA) on a visual analogue scale (VAS), and erythrocyte sedimentation rate (ESR) at baseline and 6 months. Anti-CCP titers were measured using the ImmuLisa CCP ELISA test (normal range < 25.0 U/ml, IMMCO Diagnostics Inc., USA). All patients were categorized as good or moderate/non-responders based on the EULAR response criteria. The study obtained written informed consent from all RA patients and received approval from the Institutional Review Board of Hanyang University Hospital (HYG-14-032-14). This study was performed in accordance with the relevant guidelines and regulations.

### HLA-DRB1 genotyping

We extracted DNA from the blood of enrolled patients and sequenced for HLA-DRB1 using next-generation sequencing (NGS). HLA-DRB1 SE status was defined as *01:01, *04:01, *04:04, *04:05, *04:08, *04:10, *10:01, *14:02, or *14:06. Amino acids at positions 11, 71, and 74 of HLA-DRB1 were assigned according to the sequence information provided in the IMGT/HLA Database (http://www.ebi.ac.uk/ipd/imgt/hla/), using the Sequence Alignment Tool, Release 3.53^17^.

### Statistical analyses

In a univariable analysis, we used the Wilcoxon rank sum test and chi square test for continuous (numerical) and categorical variables, respectively. A logistic regression model was used in the multivariable analyses, and odds ratios (ORs), 95% confidence intervals, and p values were estimated by a likelihood ratio test (LRT). Individuals were defined as carriers of specific alleles, amino acids, or haplotypes if they had at least one copy of the allele, amino acid, or haplotype and were tested for an association with treatment response adjusting for RA onset-age and sex. All analyses were conducted in the R environment (R 4.1.0).

### Supplementary Information

Supplementary Information.

## Data Availability

The datasets analyzed in this study are not publicly available but are available from the corresponding author on reasonable request.
