# Supplementary material for: Association of HLA-DRB1 locus with treatment response to abatacept or TNF inhibitors in patients with seropositive rheumatoid arthritis
Source: Sci Rep. 2024 Mar 21;14:6763. doi: 10.1038/s41598-024-56987-2 (PMC10957942; doi:10.1038/s41598-024-56987-2)
Supplement: Supplementary file 1 — Supplementary Information. [file 41598_2024_56987_MOESM1_ESM.pdf]

**Supplementary Table S1. Relationship between HLA-DRB1 and auto-antibodies production in seropositive RA patients**

| **HLA-DRB1** | **anti-CCP production** | | **RF production** | |
|---|---|---|---|---|
| | **OR [95% CI]** | ***P*** | **OR [95% CI]** | ***P*** |
| Shared epitope | 5.73 [2.37–14.79] | $1.1\times10^{-4}$ | 1.06 [0.45–2.30] | 0.88 |
| Valine at amino acid position 11 | 5.06 [2.07–13.58] | $3.4\times10^{-4}$ | 1.04 [0.47–2.17] | 0.93 |

HLA-DRB1 shared epitope alleles (*01:01, *04:01, *04:04, *04:05, *04:08, *04:10, *10:01, *14:02, *14:06); Valine at amino acid position 11 of HLA-DRB1 (*04:01, *04 :03, *04:04, *04:05, *04:06, *04:07, *04:08, *04:10, *10:01)

**Supplementary Table S2. HLA-DRB1 shared epitope haplotypes from amino acids at positions 11, 71, and 74**

| Pos 11 | Pos 71 | Pos 74 | Haplotype* | HLA-DRB1 shared epitope |
|---|---|---|---|---|
| Leu | Arg | Ala | LRA | *01:01 |
| Val | Lys | Ala | VKA | *04:01 |
| Val | Arg | Ala | VRA | *04:04, *04:05, *04:08, *04:10, *10:01 |

* Haplotype at amino acid positions 11, 71, and 74; Frequency of haplotype (≥1% study population frequency)
Pos, amino acid position; TNFi, TNF inhibitors; Leu, leucine; Arg, arginine; Ala, alanine; Val, valine; Lys, lysine; Ser, serine;

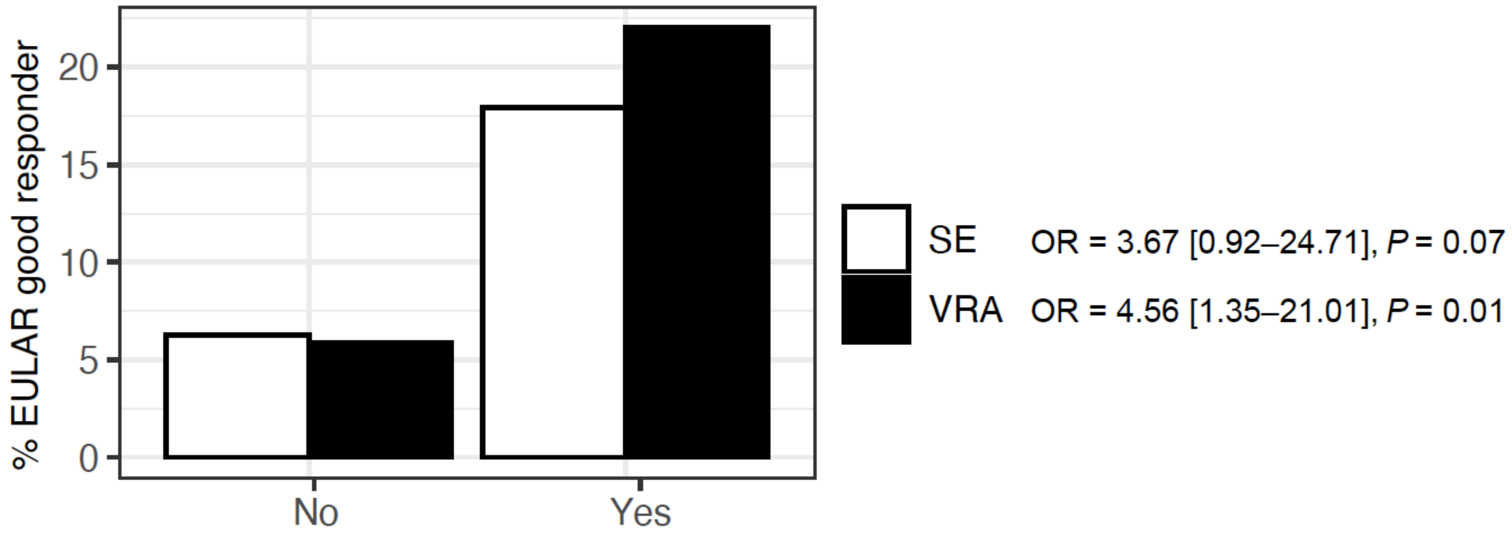


**Supplementary Figure S1. Logistic regression analysis of HLA-DRB1 SE or VRA haplotype and good responder in seropositive RA patients treated with abatacept.** Percentage of EULAR good responders based on the presence of SE or VRA haplotype. Odds ratio (OR), 95% CI, and *P* value are estimated from logistic regression analysis adjusted for RA onset age and sex.

SE, Shared epitope; VRA, Haplotype with valine, arginine, and alanine at amino acid positions 11, 71, and 74
